# Structural, Mechanical, and Transport Properties of Electron Beam-Irradiated Chitosan Membranes at Different Doses

**DOI:** 10.3390/polym10020117

**Published:** 2018-01-26

**Authors:** Alia Baroudi, Carmen García-Payo, Mohamed Khayet

**Affiliations:** 1 Department of Applied Physics I, Faculty of Physics, University Complutense of Madrid, Avda. Complutense, s/n, 28040 Madrid, Spain; abaroudi@ucm.es (A.B.); mcgpayo@fis.ucm.es (C.G.-P.); 2 Madrid Institute for Advanced Studies of Water (IMDEA Water Institute), Avda. Punto Com n° 2, Alcalá de Henares, 28805 Madrid, Spain

**Keywords:** chitosan, glutaraldehyde, electron beam irradiation, molecular structure modification, molecular weight reduction, drug release application

## Abstract

Chitosan powder irradiated by electron beam at different doses, up to 250 kGy, was used to prepare membranes for drug release applications. The irradiation effect on the molecular weight of powder chitosan, the characteristics of the prepared membranes, and their transport of sulfamerazine sodium salt (SULF) were investigated. The effect of the addition of glutaraldehyde (GLA) as a crosslinking agent in the chitosan solution used for the preparation of the membranes was also studied. A decrease in the chitosan molecular weight with the increase in the irradiation dose was observed, while the membranes prepared with the irradiated chitosan at higher dose exhibited lower swelling. However, an opposite behavior was detected when the membranes were prepared with GLA-crosslinked chitosan. A GLA crosslinking agent reduced the crystallinity of the chitosan membranes and the swelling, whereas the water contact angle and SULF transport increased with the increase in the irradiation dose.

## 1. Introduction

Chitosan is a polysaccharide polymer of natural origin composed of long chains of acetylated and deacetylated units of glucosamine linked by glucosidic bonds. The main source of obtaining chitosan is through the alkaline deacetylation of chitin. This is abundant in nature principally in shells of crustaceans, terrestrial invertebrates, and fungi [1,2]. Chitosan is a relatively weak base (p*K*a ~ 6.2) soluble in diluted organic acids with a pH < 6, such as acetic acid, that positively charge the amino groups of the chitosan [3,4]. Additionally, chitosan contains a large number of reactive –NH_2_, so it has been used extensively as an adsorbent to treat water contaminated with dyes, heavy metal ions, and other contaminants [5,6,7]. Currently, it is also used in a wide range of applications such as in food, drug, and pharmaceutical fields owing to its non-toxicity, biodegradability, biocompatibility, and antibacterial properties [2,8].

Encapsulated or prepared as a membrane, both pure chitosan and chitosan cross-linked and/or mixed with other polymers have been found as a controlled drug release barrier, since the rate of release of a substance can be controlled by determining the properties of the polymer, the manufacturing conditions, the crosslinking agents, etc. [9] and the medium in which it is to be released [10]. To perform permeation studies with chitosan membranes, its cross-linking is important [11]; due to the hydrophilic nature of chitosan, crosslinking can prevent a rapid swelling of the membrane, therefore releasing the drug immediately into the medium [12].

**Figure 1:**
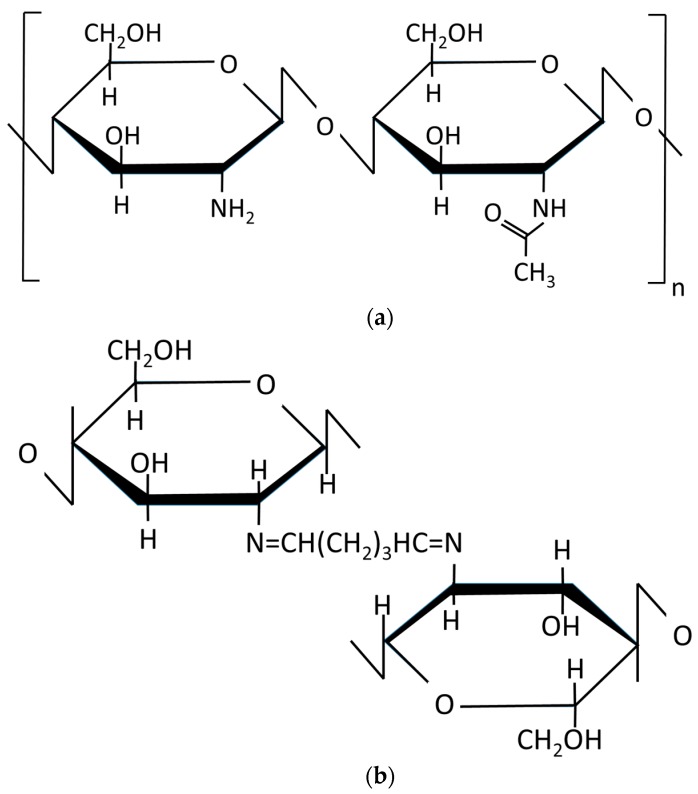
Structure of (**a**) chitosan and (**b**) glutaraldehyde (GLA)-crosslinked chitosan [17].

Covalent cross-linking agents of chitosan such as glutaraldehyde (GLA) have been commonly used to improve the functionalities of chitosan. The chemical structure of chitosan and the covalent interaction between chitosan and GLA crosslinking agent are shown in Figure 1. GLA produces Schiff bases between the aldehyde groups and the free amine groups in deacetylated chitosan polymers [2,5,13,14]. GLA reduces membrane swelling [5,15], improves its mechanical properties [2], fixes its structure, and modifies the permeation. The use of GLA with chitosan has been widely used in various research studies either as a crosslinking agent added in the chitosan solution used subsequently for the preparation of the membrane [14] or as a post-treatment of the so-prepared chitosan membrane or hydrogel [5,13,14,16].

The molecular weight (*M*_w_) and the degree of deacetylation (*DD*) are the most important physicochemical properties of chitosan. These depend on the process of deacetylation of chitin and the origin of extraction of chitin [1]. A higher *DD* makes the chitosan more biocompatible, while a lower *DD* makes the chitosan more biodegradable [1]. Moreover, chitosan with a low *M*_w_ and a high *DD* shows good solubility in physiological pH environments and exhibits a better antibacterial character than chitosan with a high *M*_w_ [18,19]. It is worth mentioning that the properties of the prepared membranes using different industrial chitosan powders depend on the *M*_w_ and the *DD*. Nunthanid et al. [20] observed that the membranes prepared with high-*M*_w_ chitosan exhibited improved mechanical properties. Jaworska et al. [21] verified that chitosan obtained from shrimp, squid, and fungi sources under the same conditions had different *DD* values and different crystallinities. Gupta and Jabrail [11] reported that the *M*_w_ and *DD* of chitosan had a significant effect on the release of centchroman from GLA-crosslinked chitosan microspheres. Therefore, a tailored chitosan with given *M*_w_ and *DD* values is required for each application.

There are different ways to reduce the molecular weight of chitosan, such as thermal degradation, acid depolymerization, enzymatic hydrolysis, oxidative degradation, electrochemical process, sonication, and swirling cavitation. However, treatments with enzymes or acid solutions induce environmental problems and have a high cost. Other methods such as ultrasonic [22] and microwaves are green but are less effective than high energy radiation (i.e., ultraviolet (UV) radiation, gamma rays, proton beam, and electron beam). High energy irradiation a provides cost reduction and environmental alternatives to change the physical, chemical, and/or biological characteristics of a product without requiring a catalyst or a temperature increase [23,24,25,26,27,28].

Chitosan can be irradiated before its use in powder form [28,29,30] or in aqueous solution [31,32] or after its use as a membrane or microsphere [33]. Among other benefits, the irradiation of chitosan increases its antimicrobial activity as well as its antitumor, antioxidant, and hypocholesterolemic properties [18,23,31]. The effect of irradiation on pure chitosan powder is a reduction in its *M*_w_ while its biocompatibility is not sacrificed [33]. In fact, irradiation induces the formation of carboxyl groups and the breakdown of glycosidic bonds, which causes shortening of the chitosan chains [34,35,36]. Another effect of irradiation is the deamination [34], although no significant variation in the *DD* was observed [31,36]. In addition to a reduction in the *M*_w_ of chitosan, a reduction in the mechanical properties of the membranes prepared with irradiated chitosan, depending on its intensity and dose [28], and a reduction in the hydrophilic character of chitosan and its absorption of water [37] were also found.

The most common techniques applied for the irradiation of polymers are γ-irradiation (γ-rays) and electron beam (e-beam) because of their short processing time, low equipment cost, and small temperature increase in the sample [31,34]. The result of the irradiated polymer by means of both techniques is similar, although for small samples the use of e-beam is more effective, because it presents higher dose rates and less exposure time, reducing the effects of oxygen degradation [30]. The long residence time allows for the continuous diffusion of oxygen in the sample, resulting in an increase in the deterioration of the properties of the material [38].

In the present study, chitosan powder was irradiated by applying an e-beam technique at different doses in order to obtain chitosan of different molecular weights. Various chitosan membranes were prepared, and their properties as well as their potential for use as a drug delivery barrier were studied. The membranes were fabricated without and with a GLA-crosslinking agent. The addition of GLA was considered for the improvement in the properties of the membranes fabricated with low-molecular-weight chitosan and to reduce their drug permeability. The membranes were characterized in terms of their swelling, water contact angle, and mechanical properties. Finally, the membrane transport was performed using sulfamerazine sodium salt (SULF), a broad spectrum antibiotic, as the agent to be released.

## 2. Materials and Methods 

### 2.1. Materials

The commercial grade chitosan powder (Shanghai Nicechem Co., Ltd., Shanghai, China) having a high density (0.6 to 0.8 kg/L), a degree of deacetylation, as supplied by the manufacturer, of 85 to 95%, and a low viscosity (i.e., less than 200 mPa·s), was used as a biopolymer. Acetic acid (Sigma-Aldrich, Saint Louis, MS, USA, 99.8% purity) was employed to prepare the chitosan solution, and glutaraldehyde (GLA, Sigma-Aldrich) was used as a crosslinking agent. The transport experiments through the membranes were performed using aqueous solution of sulfamerazine sodium salt (SULF, Sigma-Aldrich). All other chemicals were analytical grade reagents and were employed as received.

### 2.2. Electron Beam Irradiation

The chitosan powder was irradiated in solid state using “Electron Linac”, Accelerator electronics 10-10 [39] at the Institute of Chemical Nuclear and Technology in Warsaw (Poland). The chitosan was irradiated with an energy between 5 and 10 MeV in an air atmosphere. The applied doses were 12.5, 100, and 250 kGy. The codes R0, R12, R100, and R250 were considered for the membranes prepared with non-irradiated and irradiated chitosan at the doses of 12.5, 100, and 250 kGy, respectively.

### 2.3. Membrane Preparation

The chitosan membranes were prepared by solution casting followed by solvent evaporation method. The chitosan solution was prepared by mixing 1% (*w/w*) of chitosan in 2% (*w/w*) acetic acid aqueous solution. This concentration was chosen to ensure a complete dissolution of chitosan [3]. The vessel containing chitosan solution was properly closed to minimize evaporation of acetic acid and gently stirred at room temperature for 6 h using a stirrer (Heidolph Model 2021, Schwabach, Germany) at 400 rpm.

The chitosan membranes were prepared using chitosan powder irradiated at different doses without and with GLA. The used concentration of GLA was 0.1% (*w/w*) in methanol [16]. The chitosan membranes were fabricated with different concentrations of GLA in the solution. The minimum concentration to obtain membranes of chitosan CS-250 was 0.1% (*w/w*), so this concentration was added to the chitosan solution and stirred for 90 min with a magnetic stirrer. The membrane thickness was controlled by pouring a given amount of chitosan solution on glass plates 10 cm in diameter. Finally, the poured chitosan solutions were subjected to solvent evaporation and dried at room temperature to form the membranes. It is to be noted that it was impossible to prepare the membrane with irradiated chitosan at 250 kGy without adding GLA in the chitosan solution. This membrane was spontaneously cracked in small pieces during the dry process.

### 2.4. Characterization Methods

#### 2.4.1. Viscosity, Molecular Weight, and the Irradiation-Induced Chemical Degradation of Chitosan Powder

The molecular weights of chitosan powders irradiated at different doses were determined by the capillary viscosimetry method using an Ostwald viscometer. The viscosity measurements were carried out at room temperature (25 °C) using chitosan solutions of different concentrations. The intrinsic viscosity, [*η*], was obtained by extrapolating the reduced viscosity, $\eta_{\mathrm{red}}$, to zero concentration using the Huggins equation defined as [28,34]

(1) $$\eta_{red}=\left[\eta \right]+k_{H}{\left[\eta \right]}^{2}\cdot c$$

where *c* is the chitosan concentration, and *k*_H_ is the constant of Huggins. [*η*] is directly proportional to the viscosity average molecular weight, *M*_v_, according to the Mark–Houwink–Sakurada equation [22,28,32,34]:

(2) $$\left[\eta \right]=K\cdot M_{v}^{\alpha }$$

where *k* and α are constants related to the degree of deacetylation, *DD*, expressed as a percentage [22,32]:

(3) $$K=1.64\times 10^{-30}\times DD^{14}\frac{\mathrm{mL}}{g}$$

(4) $$\alpha =-1.02\times 10^{-2}\times DD+1.82.$$

The relationship between the viscosity average molecular weight (*M*_v_) and the number average molecular weight (*M*_n_) for lineal polymers such as chitosan was described by Flory [32]:

(5) $$\frac{M_{v}}{M_{n}}={\left[\left(1+\alpha \right)\Gamma \left(1+\alpha \right)\right]}^{1/\alpha }$$

where Γ(1+α) is the gamma function of 1 + *α*.

#### 2.4.2. Attenuated Total Reflectance-Fourier Transforms Infrared (ATR-FTIR) Spectroscopy and Degree of Deacetylation (*DD*)

The FTIR spectra of the chitosan powders as well as chitosan membranes were obtained using a spectrometer (Nicolet^TM^ model Magna-IR 750 series II, Madison, WI, USA), equipped with a detector DTGS-KBr (triglicerin sulfate deuterated with KBr window), a beam splitter KBr, and an infrared source (Ever-Glo^TM^, Nicolet Electronic Corporation, Madison, WI, USA) employing an attenuated total reflectance (model H-ATR Multiple Bounce, Spectra Tech, Madison, WI, USA) with a ZnSe crystal and 13 steps. The measurements were carried out with 128 scans in the wavelength range of 500–4000 cm^−1^ and an 8 cm^−1^ resolution. The chitosan powder samples were first prepared in pill form of KBr with a 3 mg sample per 300 mg of KBr (Sigma-Aldrich, FTIR grade). This analysis aimed to determine the modifications at the molecular scale induced in chitosan after e-beam irradiation and to calculate the *DD* [34,40]:

(6) $$DD\%=100-\frac{A_{1655}}{A_{2870}}\times \frac{100}{1.33}$$

where *A*_1655_ is used for the determination of the residual –CO–NH– groups, and *A*_2880_ corresponds to the absorbance related to the C–H band at 2880 cm^−1^, used as a reference band. Using this reference band is advantageous in that the peak intensity is significant, the band is not involved in the hydrogen bond, and water does not create any interference peaks [41].

The chitosan membranes were also analyzed by FTIR-ATR in order to determine the interaction between chitosan and GLA at different irradiation doses. The measurements were carried out directly from each chitosan membrane.

#### 2.4.3. X-ray Diffraction Spectroscopy

X-ray diffraction (XRD) patterns of chitosan powders and chitosan membranes were obtained using a diffractometer X’Pert-MPD (Philips, Almelo, The Netherlands) at the Cu K_α_ wavelength (λ = 1.54 Å). The scanning range varied from 5 to 90° in steps of 0.04°, with a scanning speed of 1 step/s. The operating conditions were 45 kV and 40 mA using an aperture of 0.15 mm.

#### 2.4.4. Other Characterization Methods for Chitosan Membranes

Membrane thickness was measured with a digital micrometer (Helios-Preisser GmbH, Gammertingen, Germany) with an accuracy of 0.001 mm. Forty measurements along different diameters were made and the thickness was determined as the mean value with its corresponding standard deviation.

The swelling degree (%*SD*) is the percentage of liquid that a membrane sample is able to absorb. This was calculated by gravimetric measurements as follows [11,22,42]:

(7) $$\%SD=\frac{W_{s}-W_{d}}{W_{d}}$$

where *W*_s_ and *W*_d_ are the weight of the swollen membrane at time *t* and the weight of the dry membrane, respectively. The membrane swelling was measured in distilled water and in SULF aqueous solution with a 15 g/L concentration. First, the samples were immersed in distilled water or in SULF aqueous solution at room temperature. At regular time intervals, the samples were removed from water or from SULF aqueous solution, dried with an adsorbing paper, and weighed on a precision balance Sartorius, which has an accuracy of ±0.0001 g. Three samples were used for each membrane.

The water contact angle (θ) of the top surface (in contact with air) and bottom surface (in contact with the glass plate) of the chitosan membranes were measured using a CAM100 device (KSV Instruments Ltd., Monroe, CT, USA) with the software CAM200usb. This enabled us to acquire images of distilled water drops deposited on the surface of the sample and to calculate the contact angle values. A Hamilton stainless steel needle allowed for the control of the volume of the drop, which was about (2.0 ± 0.1) µL. For each drop, five images were recorded over a period of 4 s. For each membrane, at least 12 water drops were considered to determine the average θ value together with its standard deviation.

The mechanical properties of the chitosan membranes were investigated by means of the measurements of their tensile strength ($\tau_{S}$), elongation at break ($\varepsilon_{b}$), and Young’s modulus (*E*) using a universal test device (Instron model 3366, Norwood, MA, USA), according to ASTM D 3379-75 specifications. The tensile test was performed at room temperature with a load cell of 50 N, an initial gauge length of 30 mm, and a cross-head speed of 5 mm/min. For each membrane, at least five samples were considered and the corresponding parameters were calculated as the average of the five registered data.

### 2.5. Drug Permeability Experiments

**Figure 2:**
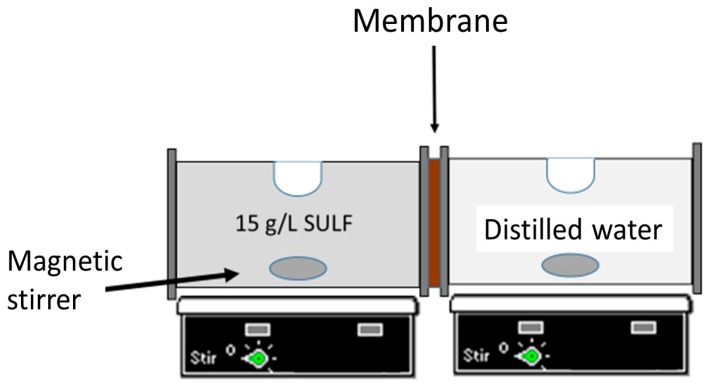
Experimental set-up used for drug permeability measurements.

The experimental device used for drug release tests consisted in two cylindrical half cells 150 mL in volume and separated from each other by the chitosan membrane, as schematized in Figure 2. The effective membrane area was 7.07 ± 0.23 cm^2^. The solution inside each cell compartment was stirred using magnetic stirrers. The left side of the cell (i.e., the feed side) was filled with a solution of 15 g/L SULF in distilled water, while the right side of the cell (i.e., the permeate side) was filled with distilled water. A UV spectrometer (UV60, Shimadzu, Kyoto, Japan) was used to analyze the SULF concentration as a function of time in both cells. Every 15 min, a 0.1 mL sample was taken from both cells and added to 2.9 mL of distilled water for UV analysis. The absorbance measurements were carried out at the wavelength λ = 261 nm, and the SULF concentration was determined by a previous calibration. 

Due to the concentration difference between the two compartments, a solute molar flux was established from the feed side filled with SULF aqueous solution through the membrane to the permeate side. This molar flux, *J_x_* (in mol/m^2^s), takes place in the *x*-axis direction normal to the membrane surface, obeying Fick’s first law [43]:

(8) $$J_{x}=-D\frac{\partial c}{\partial x}$$

where *c* is the concentration of the diffusing species, and *D* is the diffusion coefficient of the membrane. *D* is determined from Fick’s second law (Equation (9)), considering the transient state:

(9) $$\frac{\partial c}{\partial t}=-D\frac{\partial^{2}c}{\partial x^{2}}.$$

The diffusion coefficient, *D*, can be calculated using the model described by Crank [43], if it can be assumed that initially the membrane had no SULF concentration (*c*_0_ = 0), and the SULF concentration in the feed side was much higher than that in the permeate side (i.e., *c*_1_ >> *c*_2_ and *c*_1_ − *c*_2_ *≈ c*_1_). In this case, the SULF concentration at the permeate side can be expressed as a function of time (*c(t*)) by the following equation:

(10) $$c\left(t\right)=\;\frac{DAc_{1.0}}{VL}\left(t-\frac{L^{2}}{6D}\right)$$

where *A* is the effective membrane area, *c*_1.0_ is the initial concentration in the feed side, *V* is the permeate volume, *L* is the membrane thickness, and *t* is the operating time. The expression *L*^2^/6*D* is referred to the time lag, $T_{\delta }$.

The molar flux, *J*, can be also determined from the slope of SULF concentration of the permeate side versus time (*c*(*t*)/∆*t*) as

(11) $$J=\frac{c\left(t\right)V}{∆t\;A\;M}$$

where *M* is the molar mass of the SULF.

## 3. Results and Discussion

### 3.1. Chitosan Powders

**Figure 3:**
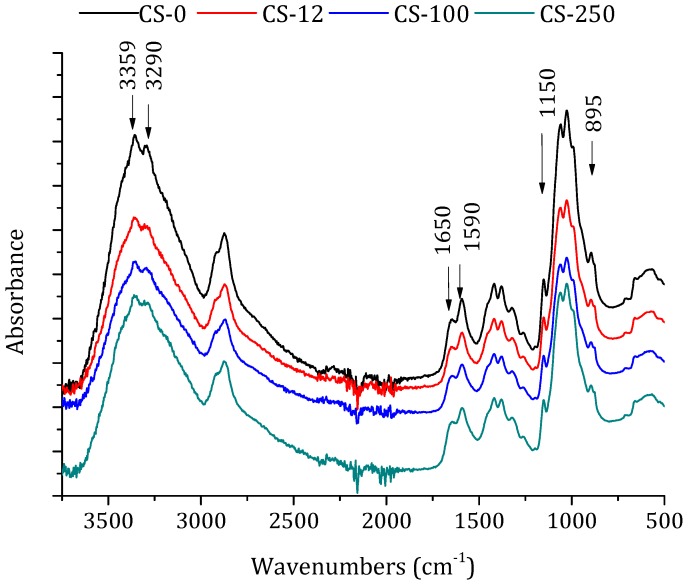
FTIR-ATR spectra of chitosan powders: non-irradiated (CS-0) and irradiated at different doses in solid state, 12.5 kGy (CS-12), 100 kGy (CS-100), and 250 kGy (CS-250).

FTIR-ATR spectroscopy was used to assess the structural changes in polymers and their derivatives after their irradiation [25]. FTIR spectra of the non-irradiated and irradiated chitosan powders at different doses are shown in Figure 3. The important characteristics bands appeared at 3359 cm^−1^ (–OH stretching), 2920, and 2875 cm^−1^ (–CH stretching vibration of pyranose ring), at 1650 cm^−1^ (stretching of C=O amide I band), at 1590 cm^−1^ (NH_2_ in amino group [44]), and at 1380 cm^−1^ (CH_3_ in amide group) [37]. The peaks at 895 and 1150 cm^−1^ were assigned to β(1-4) glycosidic bridge, and the bands 1065 and 1022 cm^−1^ to –C–O–C– bridge [25]. 

The position of the relevant peaks in the spectrum of the non-irradiated chitosan was similar to those previously described by other authors [24,25,41]. It can be clearly seen that the positions of chitosan’s characteristic bands are almost identical in the FTIR spectra of both non-irradiated and irradiated chitosan powders. Therefore, the formation of new groups was not observed due to irradiation [26], although some differences could be observed, such as the different relative intensities between 1650 and 1590 cm^−1^ (attributed to the –C=O stretching and the –C=N stretching of the amide group). A decrease in the relative intensity was detected, but without any shift, between the bands at 3359 and 3290 cm^−1^ corresponding to the loss of –OH stretching vibration. In addition, the decrease in glycosidic bond linkage (relative intensity between the bands 1150 and 895 cm^−1^) indicated the molecular chain scission of chitosan. These effects were more pronounced as the irradiation dose was increased. Similar results were observed by Benβettaïeb et al. [24] for chitosan membranes irradiated by e-beam from 20 to 60 kGy and by Gryczka et al. [30] for chitosan membranes irradiated in the dose range 30–300 kGy. Because of the overlapping of the bands corresponding to the tensions of the carboxyl groups with other bands (from 1725 to 1700 cm^−1^, from 3000 to 2200 cm^−1^, from 1420 to 1200 cm^−1^, and from 960 to 875 cm^−1^) only the change in the relative intensity could be observed. By using the electron paramagnetic resonance (EPR) technique, Gryczka et al. [30] concluded that, for e-beam irradiation doses higher than 200 kGy in the presence of oxygen (i.e., in air), stable nitroxyl-type radicals appeared as secondary products from the radiolysis of the amino group.

**Figure 4:**
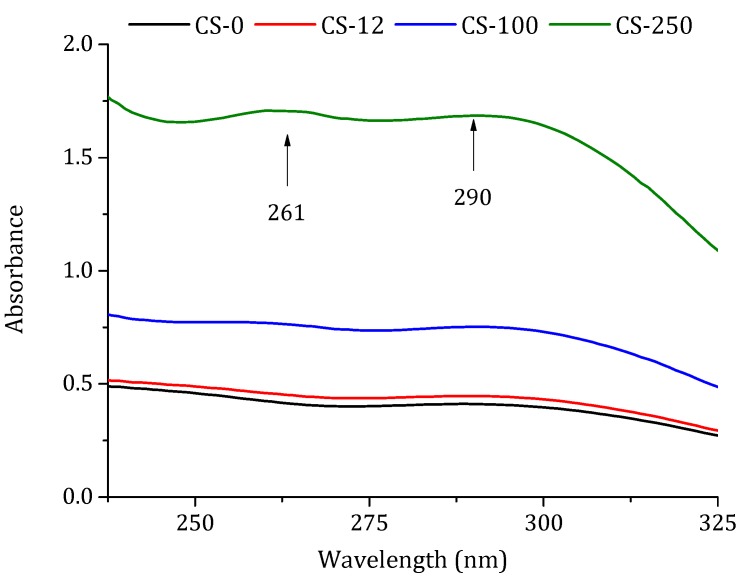
Absorption spectra of chitosan: non-irradiated (CS-0) and irradiated at different doses in solid state, 12.5 kGy (CS-12), 100 kGy (CS-100), and 250 kGy (CS-250). Measurements were taken for 1% (*w/w*) chitosan solution in 2% (*w/w*) acetic acid.

The effect of chitosan irradiation was observed in the UV spectrum, since more irradiation induced more intense absorption bands [37]. The spectra were obtained from the chitosan solution used in this work (1% (*w/w*) chitosan solution in 2% (*w/w*) acetic acid). As can be seen in Figure 4, an increase in the absorbance was observed with the increase in radiation doses, detecting broad peaks at wavelengths of 261 nm and 291 nm when the dose was 250 kGy. The absorbance of the non-irradiated chitosan CS-0 at 261 nm was 0.422, while the absorbance of the irradiated chitosan CS-12, CS-100, and CS-250 was 0.458, 0.769, and 1.707, respectively. The same behavior was observed for 291 nm, where the absorbance of CS-0 was 0.411, and that of the irradiated chitosan was 0.447, 0.753, and 1.686 for CS-12, CS-100, and CS-250, respectively. Tahtat et al. [32] found that solid-state irradiated chitosan exhibited a higher absorbance at the 261 nm band than at the 295 nm band and the increase between the absorbance and the radiation dose was not linear. The increase in the absorbance of these peaks can be attributed to the scission of the glycosidic bond linking the different chitosan chains [24,35]. Consequently, carbonyl bonds at 291 nm and carboxylic bonds at 261 nm were formed at the terminal groups of the chitosan chains, demonstrating a shortening of the chitosan chains [32].

The *DD* values calculated from the area ratio of the amide band and the C–H band (Equation (6)) obtained with FTIR measurements are summarized in Table 1. These *DD* values remained almost constant at different doses of irradiation when its corresponding errors were taken into account, with an average *DD* value of 87 ± 5%, although a slight decrease could be observed when the irradiation dose was increased (see Table 1). This result is in agreement with the reported mechanism of scission depolymerizaction of chitosan by γ-irradiation in solid state [28,45], and even by proton beam irradiation [26], where the functional groups remain unaffected by the free radicals attack.

**Figure 5:**
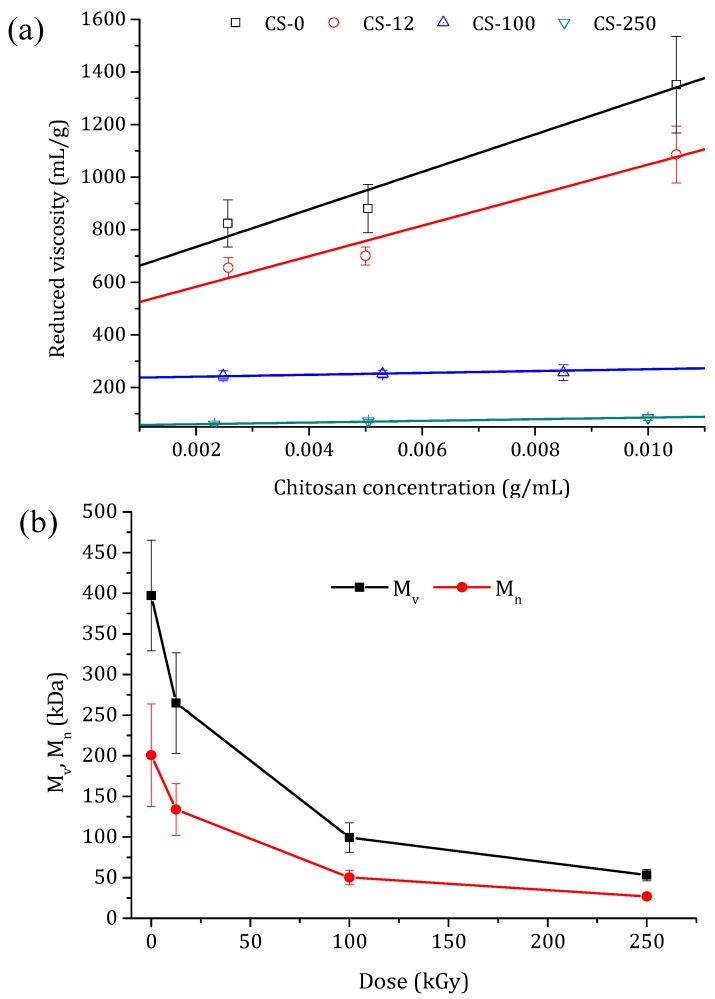
(**a**) Reduced viscosity as a function of the chitosan concentration for non-irradiated (CS-0) and irradiated chitosan powders at different doses: 12.5 kGy (CS-12), 100 kGy (CS-100), and 250 kGy (CS-250). (**b**) Change in the viscosity average molecular weight, *M*_v_, and the number average molecular weight, *M*_n_, of the chitosan powders with the applied irradiation dose.

A better explanation of the chain scission is the change in the molecular weight as a function of the irradiation dose. The reduced viscosity values with the chitosan concentration for the non-irradiated chitosan powder and the irradiated chitosan at different doses are shown in Figure 5a. By increasing the irradiation dose, a less dependence of chitosan concentration on the reduced viscosity was observed. The intrinsic viscosities, [*η*], were calculated from the intercept of the linear fits in Figure 5a, and the obtained values are shown in Table 1. With the increase in the irradiation dose, [*η*] was drastically reduced due to the cleavages of glycosidic bond and chain scissions. Taking into account the calculated *DD* values (Table 1), and by using Equations (3)–(5) (where the constant values of *K* and α are $\left(2.3\pm 0.4\right)\times 10^{-3}$ mL/g and α=0.93 ±0.01, respectively), the viscosity average molecular weight (*M*_v_) and the number average molecular weight (*M*_n_) were determined for the chitosan powder irradiated at different doses. The obtained values are also summarized in Table 1 and presented in Figure 5b. Both molecular weights decreased sharply up to 100 kGy, whereas beyond this dose slight reductions of both *M*_v_ and *M*_n_ were detected. This behavior can be attributed to the fact that drastic degradation occurred in the amorphous regions of the chitosan, and slow degradation at higher doses occurred in the crystalline regions [46]. A similar tendency was obtained by Chmielewski et al. [29], who studied the e-beam irradiation of chitosan powder (*M*_v_ = 70 kDa) within the dose range of 20–250 kGy, registering a decrease in *M*_v_ by about ten times. In comparison, the reduction in both molecular weights was lower when chitosan powder was irradiated by γ-ray irradiation as opposed to e-beam irradiation [30,32,45].

Pasanphan et al. [45] observed a decrease in the molecular weight of chitosan powder by 3 times compared to the original one when it was irradiated up to 100 kGy. Tahtat et al. [32] reported that the chitosan powder with an original *M*_v_ value of 471 kDa decreased by almost 2 times when the chitosan was irradiated up to 250 kGy. It must be pointed out that, for chitosan powder, chain scissions are mainly due to the direct effect of ionizing radiation. However, irradiation doses above 100 kGy are needed to obtain chitosan with a low molecular weight [30].

Another noticed characteristic related to the increase in the irradiation dose was the intensification of the color of the chitosan solution and the prepared dried chitosan membrane. This change in color was due to the cleavage of the chitosan chains that produce an increase in carbonyl groups [23,28] and due to the production process of chitosan [23].

### 3.2. Characterization of Chitosan Membranes

The thickness of the chitosan membranes prepared with the chitosan powder irradiated at different doses with and without GLA were measured and then related to the mass of the deposited chitosan solution on the glass plates. Based on the obtained relationship, all membranes were prepared in this study with a thickness of 35 ± 3 μm.

It was observed that the thickness of the chitosan membranes prepared with GLA were thinner than those prepared without GLA but with the same mass of chitosan. This was attributed to the crosslinking effect between GLA and chitosan as will be explained later on. The thickness reduction was greater when the irradiation dose was increased (2%, 7%, and 10% for R0, R12, and R100 membranes, respectively). Bigi et al. [47] reported that the thickness in the gelatin film decreased significantly when GLA was used as a crosslinking agent.

#### 3.2.1. ATR-FTIR Spectroscopy and X-ray Diffraction

**Figure 6:**
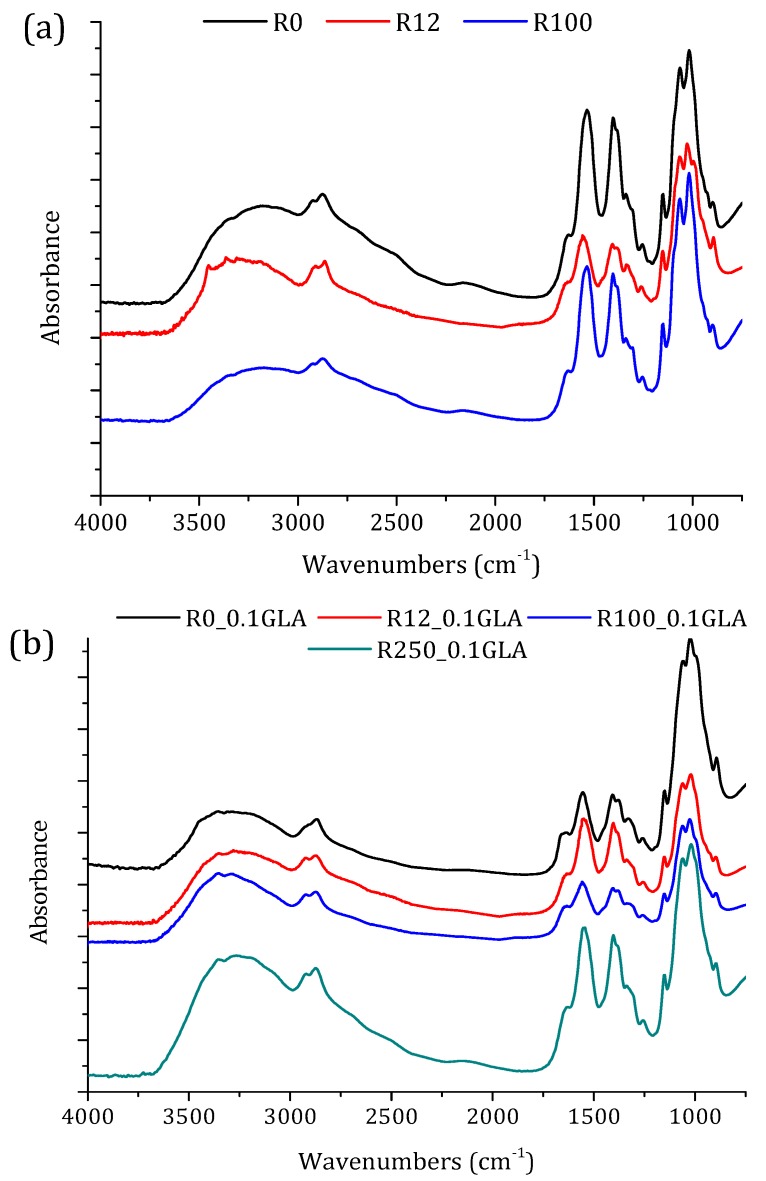
FTIR-ATR spectra of the membranes prepared with non-irradiated and irradiated chitosan at different doses: (**a**) without GLA and (**b**) with 0.1 wt % GLA in the chitosan solution.

The FTIR spectra of the prepared chitosan membranes prepared with and without GLA are shown in Figure 6. The bands at 1150 cm^−1^ (anti-symmetric stretching of the C–O–C bridge), at 1065 and 1022 cm^−1^ (skeletal vibrations involving the C–O–C stretching), and at 895 cm^−1^ are characteristic of chitosan macromolecules. All these bands appeared in chitosan membrane spectra. However, the 3359 and 3290 cm^−1^ bands corresponding to the –OH stretching vibration of chitosan powder (Figure 3) disappeared from the spectra of all chitosan membranes. The 1590 cm^−1^ band for the chitosan powder was shifted to 1534 cm^−1^ due to the influence of acetic acid [41,44]. The two new bands that appeared at 1429 and 1322 cm^−1^ correspond to –CH_2_ bending and are characteristics of *N*-acetyl glucosamine monomer [41]. From the FTIR spectra presented in Figure 3 and Figure 6a, it can be stated that the effect of the irradiation dose on the characteristic bands of chitosan was less pronounced for the chitosan membranes than those obtained for the chitosan powders, due to the reaction between the amines of chitosan and acetic acid [48].

The effect of GLA addition in the chitosan solution can be analyzed from the spectra plotted in Figure 6a,b. The membranes prepared with GLA exhibited a broader band at 1630–1650 cm^−1^ due to imine and ethylenic bonds [5,6,14,15]. On the other hand, there was no evidence of the characteristic band related to the free aldehydic group near 1720 cm^−1^. This clearly indicated that the cross-linking reaction with GLA occurred on the –NH_2_ groups of the chitosan [14]. The aldehyde groups formed covalent imine bonds with the amino groups of chitosan, due to the resonance established with the adjacent double ethylenic bonds via a Schiff reaction. Beppu et al. [5] observed that the covalent crosslinking was mainly dominated by the concentration of the covalent crosslinking agent, such as GLA, and it was favored when the molecular weight of the chitosan was increased. Moreover, since crosslinking requires mainly deacetylated reactive units, a higher *DD* of chitosan is preferred.

**Figure 7:**
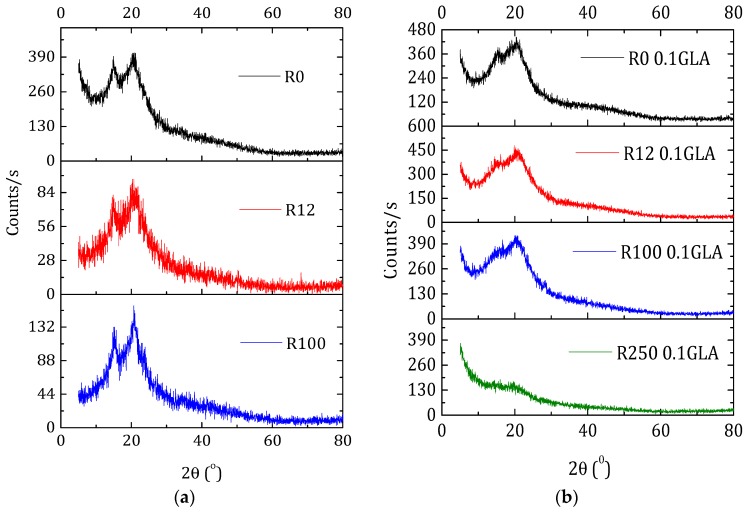
X-ray diffraction patterns of the membranes prepared with non-irradiated and irradiated chitosan at different doses: (**a**) without GLA and (**b**) with 0.1 wt % GLA in the chitosan solution.

Changes in chitosan crystallinity is important not only in terms of polymer degradation and mechanical properties, but also in terms of swelling and contact angles, which are crucial during hydration or drug transport. Figure 7a shows the XRD patterns of the membranes prepared with non-irradiated and irradiated chitosan without GLA. Besides the characteristic peak at 2θ ≈ 20°, all the chitosan membranes exhibited a second peak at 2θ ≈ 15° originated from a hydrated chitosan polymorph crystal as a complex with water and acid [49]. Compared with the membrane R0, the peak at 20° became sharper, whereas the peak at 15° became slightly broader when the irradiation dose was increased. This is partly attributed to the previously mentioned water bonding. 

The chitosan membranes prepared with GLA showed less intense and broader peaks (Figure 7b) than the chitosan membranes prepared without GLA (Figure 7a). Both peaks at 15° and 20° appeared for the GLA-crosslinked chitosan membranes irradiated up to 100 kGy. However, for the membrane R250_0.1GLA, the e-beam irradiation destructed the slight crystalline structure of chitosan and an amorphous structure was obtained. This result, which is in agreement with other previously published studies [5,27], is due to the fact that GLA was inserted into the chitosan in a previously arranged chitosan chain configuration and immobilized in the form of membrane, inducing therefore a less organized structure (i.e., amorphous copolymer).

#### 3.2.2. Swelling

**Figure 8:**
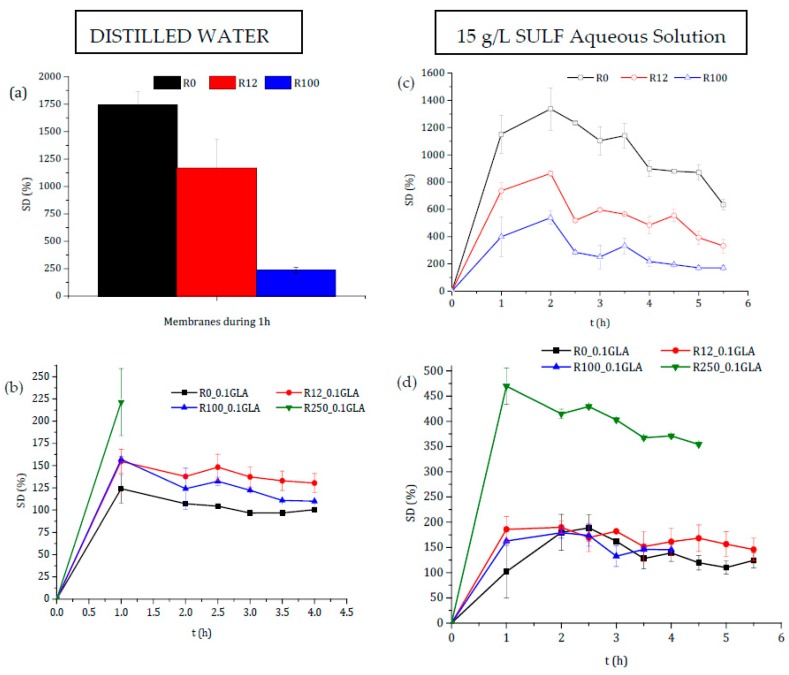
Degree of swelling in distilled water (**a**,**b**) and in 15 g/L sulfamerazine sodium salt (SULF) aqueous solution (**c**,**d**) of the membranes prepared with non-irradiated and irradiated chitosan at different doses: (**a**,**c**) without GLA and (**b**,**d**) with 0.1 wt % GLA in the chitosan solution.

The degree of swelling in distilled water of all prepared chitosan membranes without GLA is presented in Figure 8a. Swelling increased significantly during the first hour, and after that the films became gel-like and gradually dissolved into the media. This may be attributed to the higher ability of chitosan to form hydrogen bonds with water molecules, in the absence of a crosslinking agent, destroying therefore the intermolecular interactions in the chitosan chains. This behavior showed that the membranes prepared without crosslinking with GLA were not good barriers of control release, because the drug could be released very quickly in a short time due to the rapid degradation of the membrane. The higher swelling of the chitosan membranes prepared without GLA was about 10 times greater than that of the chitosan membranes prepared with GLA (see Figure 8b). This was due to the covalent imine bonds via Schiff base formation. In fact, for the chitosan membranes prepared with GLA, the degree of swelling reached the maximum in 1 h, after which it became almost constant up to 4 h reaching an asymptotic value except for the membrane R250_0.1GLA. Covalent crosslinking was influenced mainly for the *DD* of chitosan, since crosslinking required mainly the deacetylated reactive units [2]. Therefore, due to the slower relaxation time of the polymeric chains, the higher the crosslinking power is, the lower the swelling ability of chitosan membranes is, and the slower the drug release will be.

It was demonstrated that swelling depended on the *M*_w_ of the polymer chains, whereas the *DD* remained practically constant [22]. However, different trends have been reported depending on the chitosan (nature, *M*_w_, *DD*, etc.), the crosslinking agent, the blend polymers, or even the range and type of irradiation, [11,50,51]. Figure 8a,b show that the behavior depends on whether or not the chitosan membranes have been prepared with GLA-crosslinked chitosan. 

The decrease in *SD* values after irradiation for the membranes prepared without GLA was attributed to the dissolution of the hydrophilic chitosan with shorter molecular chain produced by its degradation under irradiation [51]. Zhao et al. [50] observed that the swelling of poly(vinyl alcohol) (PVA)/carboxymethylated chitosan (CM-chitosan) hydrogels decreased with the e-beam irradiation dose, but it was increased when the CM-chitosan content was increased in the blend due to the high hydrophilicity of CM-chitosan. Similar results for non-crosslinked polysaccharides membranes were obtained by Hussain et al. [42] who reported that the swelling power of the irradiated bean starches was dose-dependent and decreased significantly when the γ-irradiation dose was increased from 5 to 25 kGy.

The covalent crosslinking was favored when the *M*_w_ of the chitosan was increased (i.e., the irradiation dose was decreased), resulting in the lowest swelling ability for the R0_0.1GLA membrane [5]. This is because reinforcement between the amine bonds of chitosan further reduces the swelling of membranes with higher molecular weights, and the relaxation in chains is lower in chitosan with higher *DD* values [2]. Gupta and Jabrail [11] also observed a lower degree of swelling with chitosan microspheres crosslinked with GLA with higher *M*_w_ values. The chitosan microspheres with higher *M*_w_ values (2224 kg·mol^−1^) showed a linear increase in the degree of swelling over a time period of up to 70 h, after which it became almost constant, whereas the chitosan microspheres with a lower *M*_w_ (260 kg·mol^−1^) exhibited a higher degree of swelling due to the loss of chitosan mass after 20 h of swelling due to the decrease in intermolecular interactions.

The degree of swelling experiments was also carried out in SULF aqueous solution at different times of swelling for both chitosan membranes prepared with and without GLA. The obtained curves are also shown in Figure 8c,d. The *SD* values were decreased for the chitosan membranes prepared without GLA when the irradiation dose was increased, whereas the *SD* values were increased up to 2 h reaching maximum values, after which it was slightly reduced due mainly to the chitosan membrane mass loss. For the chitosan membranes prepared with GLA (i.e., R0_0.1GLA, R12_0.1GLA, and R100_0.1GLA membranes), no significant differences were detected. However, for R250_0.1GLA membranes, a maximum *SD* value was obtained at 1 h, after which it was slightly reduced due to the decrease in the interactions between chitosan chains. These results suggested that some kind of interaction between the chitosan membranes and the SULF solution might have taken place. Because of its cationic character, chitosan is able to react with polyanions, giving rise to polyelectrolyte complexes through electrostatic interactions.

It should be noted that SULF contains one amine group (–NH_2_) and one amide group (–NH–) corresponding to p*K*a_1_ (2.17) and p*K*a_2_ (6.77), respectively [52]. In a basic solution, SULF molecules are negatively charged and can interact with chitosan membranes due to its cationic character. The pH of the 15 g/L SULF aqueous solution was determined to be around 8. Therefore, an ionic crosslinking between chitosan membranes and SULF should take place.

#### 3.2.3. Contact Angle

**Figure 9:**
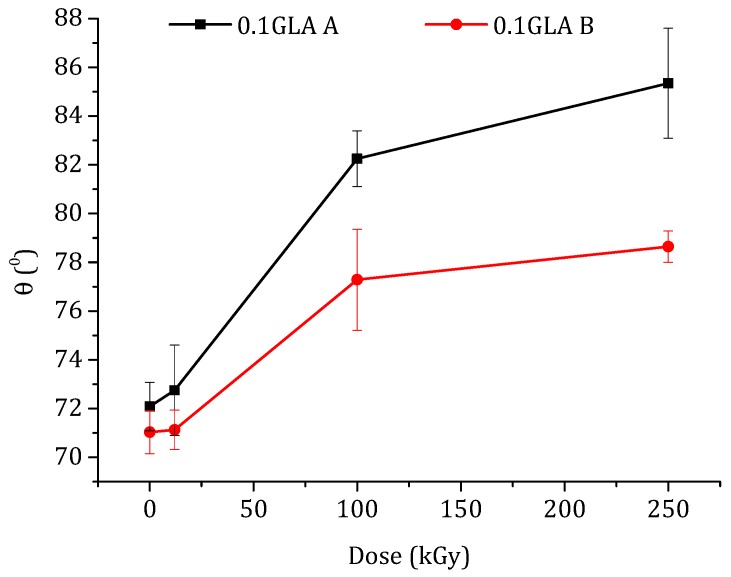
Water contact angle of the prepared membranes with GLA-crosslinked chitosan as a function of the irradiation dose: (A) top membrane surface dried in air (black line) and (B) bottom membrane surface in contact with the glass plate.

The water contact angle, θ, is an indicator of the surface hydrophilicity or hydrophobicity of a polymeric membrane. The contact angle measurements were carried out only for the chitosan membranes prepared with GLA because the water drops deposited on the chitosan membranes prepared without GLA were immediately adsorbed by the membrane due to their higher water swelling. The water contact angle values measured on both the top (i.e., dried in air) and bottom surfaces (i.e., in contact with the glass plate) of the membranes are presented in Figure 9. It is well known that the contact angle is higher for rougher surfaces than for smoother ones [53]. The plotted curves in Figure 9 show different contact angle values of the top and bottom surfaces depending on their roughness. For the same membrane, the θ values of the top membrane surface were greater than those of the bottom surfaces due to the roughness effect. This difference is more pronounced at higher e-beam irradiation doses. Kurek et al. [54] also observed higher contact angles at the air side than at the glass side (88° and 77°, respectively) of the membranes prepared with chitosan, with an *M*_w_ of 165 kDa, and glycerol (at 30% (*w/w*) of chitosan), which was used to improve the mechanical properties of the membrane.

As can be seen in Figure 9, the water contact angles increased with the increase in the e-beam irradiation dose. It is worth mentioning that the effect of e-beam irradiation on the hydrophobicity of the chitosan membrane has rarely been studied. Benβettaïeb et al. [55] evaluated the influence of the e-beam irradiation on the surface hydrophobicity of gelatin membranes and found that the water contact angle values decreased from 54.3 to 51.3° when the gelatin membrane was irradiated at 60 kGy. Shahbazi et al. [56] investigated the effect of different e-beam doses (10, 20, 30, and 40 kGy) on the water contact angle of chitosan/clay (cloisite 20A) nanocomposite membrane. It was observed that the water contact angle of this membrane increased up to 30 kGy and then drastically decreased at 40 kGy. Aranaz et al. [1] reported that a higher degree of crosslinking of the chitosan membranes reduced their hydrophobicity. These reported results and observations are in good agreement with the results obtained in the present study. It can be concluded that, for a higher irradiation dose, lower GLA-crosslinking power may occur because *M*_w_ is lower, obtaining higher θ and *SD* values.

#### 3.2.4. Mechanical Properties

The mechanical properties of the prepared membranes with non-irradiated and irradiated chitosan at different doses, with and without GLA used as a crosslinking agent, are summarized in Table 2. Young’s modulus (*E*), tensile strength (τ_s_), and elongation at break (ε_b_) of all studied chitosan membranes decreased with the increase in the irradiation dose. This may be attributed to the reduction in the *M*_w_ of the chitosan and its molecular chain degradation when the irradiation dose was increased, obtaining shorter polymer chains. A clear improvement in all mechanical properties was observed when the membranes were prepared with GLA-crosslinked chitosan. The values of *E*, τ_s_, and ε_b_ of the chitosan membranes prepared with GLA were higher than those of the prepared membranes without GLA. This is due to the covalent imine bonds established between the aldehyde groups of the GLA and the amino groups of chitosan, with the adjacent double ethylenic bonds via Schiff reaction that strengthen the chitosan chains. 

It is worth mentioning that Zainol et al. [28] also observed a decrease in both τ*_s_* values (i.e., from 15.33 to 7.0 MPa) and ε_b_ values (i.e., from 63.96 to 11.41%) of the membranes, prepared with 4% (*w/v*) chitosan in 2% (*v/v*) acetic acid without any surfactant, with the increase in the γ-ray irradiation dose applied to chitosan powders up to 100 kGy. The higher τ_s_ and ε_b_ values obtained by Zainol et al. [28] compared to those observed in the present study demonstrate the effect of chitosan concentration on these parameters. However, García et al. [23] reported that the τ_s_ values increased, while the ε_b_ values decreased as the irradiation dose increased up to 50 kGy for chitosan membranes prepared with 1.5 and 2% (*w/v*) chitosan powder in a solution of lactic acid using 1% (*v/v*) and 0.1% (*v/v*) of Tween 80. However, Li et al. [57] observed an increase in both τ_s_ and ε_b_ with the increase in γ-ray irradiation doses up to maximum values of 25 kGy; for doses higher than 25 kGy, up to 80 kGy, a sharp decrease in both parameters was registered.

For e-beam irradiation, Benβettaïeb et al. [24] reported that the τ_s_ values of gelatin membranes increased significantly with the irradiation dose (i.e., improved by 30% for 60 kGy); however, for chitosan membranes, it was not significantly affected. In contrast, the irradiation reduced the ε_b_ values of the chitosan membranes to 50% and enhanced the *E* values by two times. In another study [58], it was stated that the τ_s_ values of a chitosan-fish gelatin membrane increased sharply when the irradiation dose was increased up to 60 kGy. The main reason for these observed differences might be the different preparation methods of the chitosan membranes.

### 3.3. Drug Permeability Experiments

**Figure 10:**
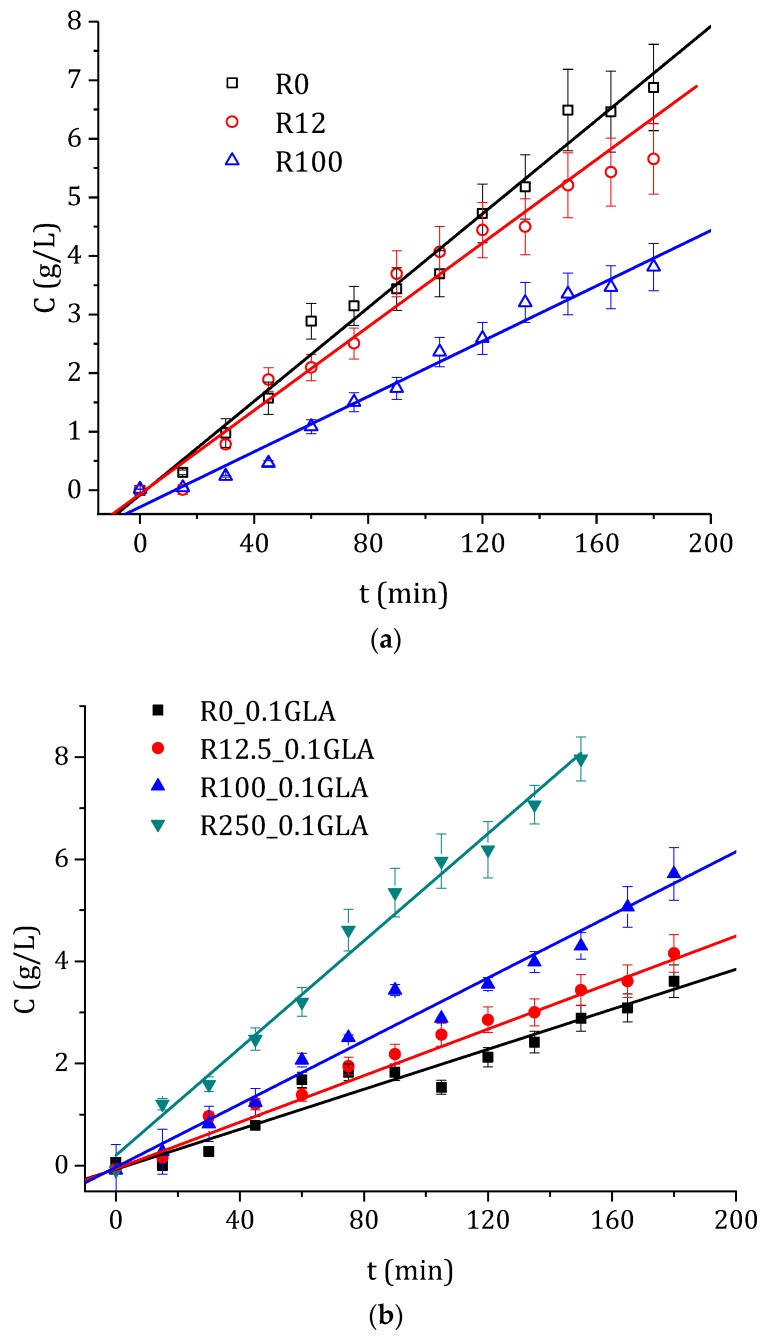
Temporal evolution of the concentration of SULF in the permeate side of the membranes prepared with non-irradiated and irradiated chitosan at different doses, (**a**) without and (**b**) with GLA in the chitosan solutions.

In order to compare SULF permeation experiments of the prepared chitosan membranes, the concentration of SULF in the permeate side is plotted in Figure 10 as a function of the operating time. As was expected, a linear dependence of the SULF concentration with time was observed with reasonably good R-squared coefficients (greater than 0.94 in all cases). Consequently, a Fickian linear approximation could be made. Therefore, the diffusion coefficient, *D*, and the time lag, *T*_δ_, were evaluated from the linear fit as is indicated by Equation (11). Additionally, the permeate molar flux, *J*, was also determined by means of Equation (12). The obtained values of *D*, *T*_δ_, and *J* are also summarized in Table 2 for all tested membranes. 

As can be seen in Figure 10 and Table 2, again an opposite behavior was detected for the chitosan membranes prepared without and with GLA. The diffusion coefficient decreased with the increase in the e-beam irradiation for the chitosan membranes prepared without GLA, whereas it was increased for the chitosan membranes prepared with GLA. The same trends were observed for the molar flux. These results are in agreement with the swelling data shown in Figure 8. The chitosan membrane had a higher drug permeation and exhibited a higher degree of swelling [59]. For the time lag values, in general, opposite behavior was observed in the chitosan membranes prepared with and without GLA. By increasing the e-beam irradiation dose, *T*_δ_ was increased in the membranes prepared without GLA in the chitosan solution, whereas it was decreased in the membranes prepared with GLA. The membranes prepared with chitosan of greater molecular weight, less swelling, less amorphous structure, and smaller hydrophobicity resulted in a reduced penetration of the drug into the membrane and a slower drug release as a consequence [1,59]. These facts might be because the relaxation time of the polymer chains decreased, requiring therefore more time for the drug to penetrate the membrane [2] and a long period of time for the stationary state to be established [60].

Gupta and Jabrail [11] indicated that the *M*_w_ and *DD* of chitosan had a significant effect on the release of centchroman from GLA-crosslinked chitosan microspheres. The diffusion coefficient of centchroman from microspheres with low *M*_w_ chitosan (260 kg·mol^−1^) was found to be 2.9 × 10^−12^ cm^2^/s. It was also claimed that the diffusion was also influenced by the nature of the crosslinking agent. As has been previously mentioned, a lower-*M*_w_ chitosan led to lower GLA crosslinking power, resulting in higher swelling and a greater diffusion coefficient. Consequently, the physicochemical nature of the crosslinking agent and that of any additional polymer allow for the modulation of drug release. 

**Figure 11:**
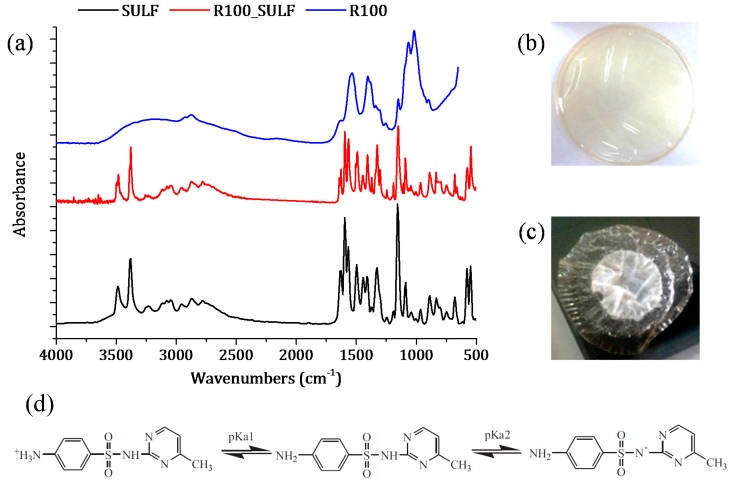
SULF sorption phenomenon of the chitosan membrane R100: (**a**) FTIR spectra of SULF and the chitosan membrane irradiated at 100 kGy before (R100) and after transport experiment (R100_SULF); images of the R100 membrane (**b**) before and (**c**) after SULF transport experiment. (**d**) Scheme illustration of dissociation equilibrium of SULF [52].

In addition, if the pH and the p*K*a values of chitosan membranes and SULF solution are taken into account, the chitosan membranes and SULF can interact ionically [61]. In fact, the pH of the membranes prepared with non-crosslinked chitosan was 3.4, while that of the membranes prepared with GLA-crosslinked chitosan was 5. SULF contains one amine group (–NH_2_) and one amide group (–NH–) corresponding to p*K*a_1_ (2.17) and p*K*a_2_ (6.77), respectively [52] (see Figure 11d). Therefore, the SULF aqueous solution (pH 8) was negatively charged, indicating that the membrane was under its protonated form and could ionically interact with SULF. The interaction between SULF and the R100 membrane is shown in Figure 11a–c.

The FTIR spectrum of the R100 membrane after the SULF transport experiment is similar to SULF spectrum, indicating that a high interaction occurred between SULF and the membrane R100. In addition, a simple visual inspection of the R100 membrane after the SULF transport experiment clearly revealed this ionic interaction (adsorption) between chitosan and SULF. It is worth noting that, among all tested chitosan membranes, the R100 membrane exhibited the lowest diffusion coefficient. This indicated that, in the absence of GLA crosslinking, most interaction between the chitosan membrane and SULF took place when the highest e-beam irradiation dose was considered as the R100 membrane exhibited the shortest chitosan chains.

The ionic interaction between chitosan membrane surface and SULF was lower for the membranes prepared with GLA-crosslinked chitosan, resulting in a diffusion coefficient higher than that of the membranes prepared with non-crosslinked chitosan. Therefore, the free radicals of chitosan formed by the chain scission during e-beam radiation might be linked to GLA. Moreover, due to the faster relaxation time of the polymeric chains, the lower the crosslinking power is (i.e., the higher the irradiation dose is), the higher the swelling ability of chitosan membranes is, leading to a sharp increase in the drug release rate [2]. This may explain the trend observed for the time lag values. The faster time lag corresponds to that of the membrane R250_0.1GLA prepared with GLA and irradiated chitosan at the highest dose.

## 4. Conclusions

Chitosan irradiation up to 100 kGy resulted in a significant reduction in its molecular weight and intrinsic viscosity [*η*], while only a slight reduction was detected for higher irradiation doses. This was attributed to the fact that low irradiation drastically reduced cleavages of glycosidic bonds in the chitosan chains in the amorphous regions, whereas a slower degradation at higher doses took place in their crystalline regions. No significant variation in the *DD* could be detected between irradiated chitosan at different doses. 

The membrane crystallinity decreased considerably when chitosan was irradiated even at 12.5 kGy. The use of the GLA crosslinking agent reduced the crystallinity of the chitosan membranes.

When the e-beam irradiation dose was increased, *SD* values decreased abruptly for the membranes prepared with uncrosslinked chitosan, while it was increased slightly for the membranes prepared with GLA-cross-linked chitosan. The decrease in *SD* values of the irradiated chitosan membranes prepared without GLA can be attributed to the dissolution of shorter molecular chain hydrophilic chitosan produced by the degradation of chitosan under irradiation. On the other hand, covalent crosslinking was less advantageous when the molecular weight of chitosan was lower, resulting in a greater swelling capacity of the membrane prepared with non-irradiated chitosan.

A linear dependence was obtained for the variation in SULF concentration with the operating time in the permeate side of the membrane. An opposite behavior was detected for the diffusion coefficient of the chitosan membranes prepared without and with GLA. The diffusion coefficient was decreased with the increase in the e-beam irradiation for the chitosan membranes prepared without GLA, whereas it was increased for the chitosan membranes prepared with GLA. The same trends were observed for the molar flux, and these results were found to be in good agreement with the swelling degree of the membranes. The membranes with higher drug permeation also exhibited a higher degree of swelling. In addition, the ionic interaction was lower for the membranes prepared with GLA-crosslinked chitosan, resulting in a higher diffusion coefficient than that of the membranes prepared with non-crosslinked chitosan. Moreover, due to the faster relaxation time of the polymeric chains, the lower the crosslinking power was, the higher the swelling ability of the chitosan membranes was, causing a sharp increase in the drug release rate. Therefore, other than the crosslinking power that depends on the molecular weight of the chitosan, the chemical interaction of the crosslinking agent allows for the modulation of drug release with diffusion coefficients varying in the range of 0.3–37 × 10^−12^ cm^2^/s.

## Figures and Tables

**Table 1:** Effect of electron beam irradiation on the degree of deacetylation, *DD*, intrinsic viscosity, [η], viscosity average molecular weight, *M*_v_, number average molecular weight, *M*_n_, and average number of scissions, *N*.

Dose (kGy)	*DD* (%)	[η] (mL/g)	*M*_v_ (kDa)	*M*_n_ (kDa)	*N*
0	88 ± 6	344 ± 172	367 ± 77	186 ± 39	-
12.5	87 ± 6	247 ± 104	256 ± 50	130 ± 26	0.2 ± 0.2
100	86 ± 6	87 ± 19	83 ± 13	42 ± 7	2.72 ± 0.04
250	85 ± 3	47 ± 5	43 ± 6	22 ± 3	6.11 ± 0.02

**Table 2:** Mechanical properties (Young’s modulus, *E*; tensile strength, $\tau_{S}$), and transport parameters (diffusion coefficient, *D*; molar flux, *J*; time lag, *T*_δ_) of the membranes prepared with non-irradiated and irradiated chitosan at different doses, without and with 0.1 wt % GLA in the chitosan solution. The drug permeability tests were carried out using 15 g/L SULF aqueous solution.

Membrane	*E* (MPa)	τ_s_ (MPa)	ε_b_ (%)	*D* (10^−12^ cm^2^/s)	*J* (10^−4^ mol/m^2^s)	*T*_δ_ (min)
R0	2410 ± 650	62 ± 9	5.0 ± 0.4	1.74 ± 0.07	3.3 ± 0.1	1.9 ± 0.1
R12	2270 ± 340	56 ± 9	4.1 ± 0.7	1.52 ± 0.09	3.0 ± 0.2	2.2 ± 0.2
R100	1520 ± 180	20 ± 9	1.5 ± 0.8	0.3 ± 0.1	1.95 ± 0.08	12.1 ± 0.7
R0_0.1GLA	3140 ± 490	75 ± 5	11 ± 2	2.0 ± 0.2	1.6 ±0.5	1.7 ± 0.2
R12_0.1GLA	2670 ± 230	62 ± 15	8 ± 3	2.3 ± 0.1	1.9 ± 0.1	2.5 ± 0.2
R100_0.1GLA	2290 ± 150	47 ± 18	3 ± 1	3.8 ± 0.1	2.6 ± 0.1	0.9 ± 0.07
R250_0.1GLA	2140 ± 150	37 ± 8	2 ± 1	37 ± 1	4.7 ± 0.2	0.09 ± 0.04
